# Dietary Supplements as a Major Cause of Anti-doping Rule Violations

**DOI:** 10.3389/fspor.2022.868228

**Published:** 2022-03-25

**Authors:** Fredrik Lauritzen

**Affiliations:** Science and Medicine, Anti-doping Norway, Oslo, Norway

**Keywords:** doping, dietary supplements, multi-ingredient pre-workout supplements, stimulants, athlete, team sport, prohibited substances

## Abstract

Dietary supplements encompass a large heterogenic group of products with a wide range of ingredients and declared effects used by athletes for a multitude of reasons. The high prevalence of use across all sports and level of competition, combined with the well-documented risks of such products containing prohibited substances have led to several doping cases globally. Despite being a considerable concern and persistent focus of sport organizations and anti-doping agencies, the magnitude of anti-doping rule violations associated with supplement use is not well-known. This study examines 18-years of doping controls of a national anti-doping program to determine the relationship between the presence of prohibited substances in athlete's doping samples and the use of dietary supplements. In 26% (*n* = 49) of all the analytical anti-doping rule violation cases in the period 2003–2020 (*n* = 192), the athlete claimed that a dietary supplement was the source of the prohibited substance causing an adverse analytical finding. Evidence supporting this claim was found in about half of these cases (*n* = 27, i.e., 14% of all analytical ADRV's). Stimulants were the most prevalent substance group linked to supplements (*n* = 24), of which methylhexanamine was associated with 16 cases. High risk products were predominantly multi-ingredient pre-workout supplements (*n* = 20) and fat-burning products (*n* = 4). Anti-doping organizations should develop strategies on how to assist athletes to assess the need, assess the risk and assess the consequences of using various dietary supplements.

## Introduction

To protect the right of athletes to participate in doping free sport and to harmonize anti-doping regulations in all sports and in all countries across to world, the World Anti-Doping Agency (WADA) has developed a set of rules and mandatory elements known as the World Anti-Doping Code (WADC), of which the world anti-doping program is based. Among the main purposes of the WADC is to define actions by athletes which constitute anti-doping rule violations (ADRV's), and which may trigger consequences or sanctions to the athlete. Approximately 80% of all anti-doping offenses in global sports are related to WADC article 2.1: The presence of a prohibited substance or its metabolites or markers in an athlete's urine or blood sample. The substances considered as banned for use by athletes in- and out of competition and in specific sports are regulated by the annually updated WADA Prohibited list.

Violations of WADC article 2.1 are often related to deliberate and carefully planned use of a prohibited substance with the aim of increasing athletic performance and thus get an unfair advantage over other athletes (Morente-Sanchez and Zabala, 2013). However, in some cases, the athlete tests positive for a prohibited substance after inadvertently having consumed a banned substance by eating contaminated food or using a dietary supplement containing a substance on the WADA Prohibited List (Yonamine et al., 2004; Chan et al., 2016, 2019; Walpurgis et al., 2020). This could have detrimental consequences for athletes, as the principle of strict liability of the WADC means that each athlete is liable for the substance found in his or her bodily specimen, whether or not the athlete used the substance intentionally.

Between 40 and 100% of athletes use dietary supplements, depending on country, type of sport, athlete level and definition of supplements (Garthe and Maughan, 2018). Athletes use supplements for a multitude of reasons, such as to support basic nutritional needs, manage nutritional deficiencies, improve and/or sustain health and enhance physical performance (Garthe and Maughan, 2018).

Undeclared prohibited substances in dietary supplements have been recognized for more than two decades (Geyer et al., 2000; Ayotte et al., 2001; Baylis et al., 2001), and recent studies suggest this problem remains (Mathews, 2018; Duiven et al., 2021). For example, Geyer and colleagues found that of 634 non-hormonal supplements purchased in 13 countries in 2000–2001, 15% were contaminated with anabolic-androgenic steroids not declared on the label (Geyer et al., 2004). In a more recent review, Martínez-Sanz et al. (2017) reported rates of contamination of WADA prohibited substances in ergo-nutritional supplements of between 12 and 58%. Dietary supplements intended for athletes and sports people could also be openly declared with prohibited substances (Helle et al., 2019). For anti-doping agencies, it raises concern that athletes often use dietary supplements without consulting physicians (Waddington et al., 2005; Baltazar-Martins et al., 2019) or checking the products for safety or quality (Baltazar-Martins et al., 2019), and that they are unaware of the possible risk accompanying such products (Petroczi et al., 2007).

The high prevalence of supplement use, combined with the persistent risk of contamination, have resulted in numerous warnings from WADA and national anti-doping agencies about the risk of violating WADC article 2.1 when using dietary supplements. However, since WADA does not provide details on the source of the prohibited substance in their annual ADRV reports, global statistics on the magnitude of doping from supplement use is scarce. An estimation of the scale of the problem were given by Outram and Stewart (2015) when they examined public available information provided by the national anti-doping agencies in Australia, the UK and the US, and found that 6–9% of all doping cases in these countries in the period 2005–2013 were likely attributed to use of dietary supplements.

Enhancing athlete awareness of the risk of doping when using dietary supplements has been suggested as important to reduce the incidents of such doping violations (Chan et al., 2020). The scope of this study was therefore to examine the proportion of ADRV's associated with dietary supplements by using doping test statistics and ADRV data from 18-years of doping controls by the National anti-doping agency of Norway.

## Methods

### Data Extraction and Material

Doping test statistics were extracted from Anti-doping Norway's annual reports publicly available at www.antidoping.no. All doping tests where Anti-doping Norway had been the Testing authority (i.e., The Anti-Doping Organization that authorizes testing on athletes it has authority over) from the establishment of the organization in 2003 up until the end of 2020 were included. The material included doping samples collected from Norwegian athletes performing their sport in Norway or abroad, as well as from athletes from other countries exercising their sport in Norway under the jurisdiction of a national sport federation.

Athletes were registered as Registered testing pool (RTP) athletes, National level (NL) athletes or Recreational athletes, respectively, depending on their performance level and the type of sport. RTP athletes are subject to the greatest amount of testing and are required to provide whereabout information. NL athletes compete at the highest national level, including team sport athletes competing in the top national divisions in team sports, such as football, handball, ice hockey and floorball. For individual sports, medalists in national championships in the same year or the year preceding the year of the ADRV were registered as NL athletes. At any given time in the period 2003–2020, there were approximately 140 RTP athletes and 2000–4000 NL athletes in Anti-Doping Norway's testing pool. All other athletes tested by Anti-Doping Norway during the 18-year long period were considered as Recreational athletes.

The sport and sport discipline were recorded for the athlete in each ADRV case. Sport disciplines were then divided into the following groups based on the physiological properties of the sport: Ball and team sports, Strength and power sports, Muscular endurance sports, Fighting sports, VO2max endurance sports, Gymnastic sports and Other sports.

### The Use of Dietary Supplements in Relation to ADRV's

Recorded documents on each ADRV case from Anti-Doping Norway's paper- and electronic archives were thoroughly examined to determine whether there existed a possible association between the use of a specific dietary supplement and the presence of a prohibited substance in the athlete's doping sample resulting in an ADRV. Assessments and final decisions made by Anti-Doping Norway's Prosecution Committee and the Judiciary Committee of the Norwegian Olympic Committee were used as a basis for the evaluation, and was supported by other documents recorded on each ADRV case, including correspondence and reports of conversations between Anti-Doping Norway's investigators and the athlete, support personnel, team mates and other witnesses, evidence provided by the athlete, such as pictures and/or physical samples of the dietary supplement(s) used by the athlete and which were the alleged source of the prohibited substance, expert statements from the Norwegian Doping Control Laboratory or other experts, and in some cases laboratory screening reports of the supplement(s). Furthermore, name of the supplement(s), supplement category and how/where the product was obtained by the athlete was registered for each case.

Supplements were categorized into Sports foods, Medical supplements, Ergogenic supplements and Other supplements as presented by Garthe and Maughan (2018). In addition, the groups Natural products, e.g., plants, herbs and roots, and multi-ingredient pre-workout supplements (MIPS) were added as additional categories. The latter is a new class of dietary supplements gaining increased popularity often containing a blend of ingredients with ergogenic and non-ergogenic properties, such as caffeine, creatine, beta-alanine, amino acids and nitric oxide agents (Harty et al., 2018; Jagim et al., 2019).

## Results

### Anti-doping Rule Violations 2003–2020

In the period 01.01.2003 to 31.12.2020, there were 223 anti-doping rule violations (ADRV's) from Anti-Doping Norway's testing program, where Anti-Doping Norway was the Testing authority. Of these, 86% (*n* = 192) were related to World Anti-Doping Code article 2.1, presence of a prohibited substance or its metabolites or markers in an athlete's sample (i.e., analytical ADRV's) (Table 1). All the adverse analytical findings (AAF's) were found in urine samples. Anabolic substances (WADA Prohibited list group S1) were found in 42% of the analytical ADRV cases (*n* = 81 out of 192), stimulants (group S6) in 28% of the cases (*n* = 53 out of 192) and cannabinoids (group S8) in 26% of the cases (*n* = 49 out of 192).

**Table 1:** Analytical anti-doping rule violations (ADRV's) associated with dietary supplements by year.

**Year of sanction**	**Analytical ADRV's^a^**	**Analytical ADRV's claimed to be caused by dietary supplements**	**Analytical ADRV's associated with dietary supplement use^b^**
		***n* (% of total analytical ADRV's)**	***n* (% of total analytical ADRV's)**
2003	5	2 (40%)	1 (20%)
2004	11	1 (9%)	1 (9%)
2005	11	2 (27%)	0 (0%)
2006	7	2 (29%)	0 (0%)
2007	11	2 (18%)	0 (0%)
2008	8	2 (25%)	2 (25%)
2009	8	1 (13%)	0 (0%)
2010	18	3 (17%)	1 (6%)
2011	14	3 (21%)	3 (21%)
2012	14	7 (50%)	5 (36%)
2013	7	2 (29%)	1 (14%)
2014	15	1 (7%)	0 (0%)
2015	8	3 (38%)	3 (38%)
2016	15	2 (13%)	0 (0%)
2017	6	0 (0%)	0 (0%)
2018	12	7 (58%)	4 (33%)
2019	17	7 (41%)	5 (29%)
2020	5	2 (40%)	1 (20%)
Total	192	49 (26%)	27 (14%)

a *Anti-doping rule violations related to World Anti-Doping Code article 2.1; Presence of a prohibited substance or its metabolites or markers in an athlete's urine sample*.

b *Cases were there were found evidence supporting that it was likely that a dietary supplement contained a prohibited substance corresponding to what was found in the athlete's urine sample*.

### Anti-doping Rule Violations Associated With Dietary Supplements

In 49 (26%) of the 192 analytical ADRV cases, the athlete claimed that the use of one or more specific dietary supplements must have contained a prohibited substance that resulted in the AAF (Table 1). More than half (57%) of these 49 cases (*n* = 28) were related to stimulants, whereas 17 (35%) cases were related to anabolic substances. There were no examples of athletes who tested positive for Cannabinoids only that explained the AAF by supplement use.

Evidence supporting a causal relationship between the use of a specific dietary supplement and the prohibited substance detected in the athlete's urine sample were found in 27 of the 49 cases (Table 1). In additional nine cases, the Prosecution Committee or the Judiciary Committee did not exclude that a dietary supplement used by the athlete had contained a prohibited substance resulting in the AAF, but the athlete was not able to prove this sufficiently. Taken together, the proportion of analytical ADRV's attributed to the use of dietary supplements containing prohibited substances likely lays between 14 and 19% (27–36 og 192 cases) of all analytical ADRV's in the 18-year long period.

The proportion of ADRV's causally related to supplement use in comparison to total analytical ADRV's in a respective year ranged from 0 to 36%, with no clear trend throughout the period, although supplement ADRV's constituted a greater proportion of total analytical ADRV's in the most recent 3 year period (2018–2020), compared to the first 3 years of the period (2003–2005) (29%, *n* = 10 of 34 vs. 7%, *n* = 2 of 27) (Table 1).

For the remaining 13 cases, dietary supplements were not found to be a likely source of the prohibited substance found in the athlete's sample, in contrast with the athlete's suggestion. There were different reasons for why reliable evidence could not be established. In six of the 13 cases, the athletes reported using a wide range of specific and non-specific supplements bought from different stores, however the athlete could not provide sufficient information about all the supplements used, such as product names or manufacturer, nor could the athlete provide samples of the products for potential laboratory analysis. Among these six cases, in addition to extensive use of dietary supplements, two athletes had also used over-the counter pharmaceuticals bought in Thailand prior to the doping control. In another three cases, the athletes had used dietary supplements declared with prohibited stimulants (methylhexanamine, *n* = 2 and ephedrine, *n* = 1), however, since all three doping samples were collected out of competition, where stimulants according to the WADA Prohibited list are not prohibited, the urine samples were not analyzed for stimulants. Rather the laboratory detected two cases of anabolic steroids and one sample positive for tamoxifen (a selective estrogen receptor modulator). It was not found a probable association between these substances and the dietary supplements used by the athletes. In two cases, the dietary supplements were sent to a laboratory for confirmation analysis, but the prohibited substances found in the athlete's urine sample were not detected in the supplement. In one case the athlete claimed to have received an unspecific energy drink from a friend but could not provide any other supporting information. In the last case, the athlete who tested positive for cocaine claimed to have used a natural product made from the coca plant. He could, however, not provide any information supporting this.

### High Risk Supplements and Prohibited Substances Found in Dietary Supplements

Stimulants were by far the most prevalent substance group linked to supplements containing prohibited substances, constituting 89% (*n* = 24 of 27) of all the cases (Table 2), of which the stimulant methylhexanamine was associated with 16 of the 27 cases. Other stimulants associated with dietary supplements were ephedrine (*n* = 2), sibutramine (*n* = 2), oxilofrine (*n* = 1), 4-methylpentan-2-amine (*n* = 1) and n-etyl-1-fenylbutan-2-amine (*n* = 1). Furthermore, two ADRV cases were related to dietary supplements with anabolic substances and one case with the beta-2 agonist higenamine.

**Table 2:** Prohibited substances associated with supplements causing analytical anti-doping rule violations (ADRV's).

**Substance group**	**Number of cases**	**Prohibited substances detected in athlete's biological sample**	**Number of cases**
S1	2	Anabolic substances	2
S3	1	Higenamine	1
S6	24	Methylhexanamine	17
		Ephedrine	2
		Sibutramine	2
		Oxilofrine	1
		4-methylpentan-2-amine	1
		N-etyl-1-fenylbutan-2-amine	1
Total	27		27

Multi ingredient pre-workout supplements (MIPS) and supplements in the Other supplement's category were found to be the likely source of the prohibited substance in 20 (74%) and 7 (25%) cases, respectively (Table 3). Of the dietary supplements in the Other supplement category, four were related to fat burning products, two to muscle building supplements and one to a product claiming to boost energy.

**Table 3:** Evidence for the use dietary supplements causing an adverse analytical finding by dietary supplement category.

**Dietary supplement category**	**Examples**	**Cases with no evidence**	**Causal relationship not established but probable**	**Cases with evidence**
Sports foods	Protein drinks, protein powder, gainer, sports gel, sports drink, energy bars etc.	1	0	0
Medical supplements	Vitamins, minerals, fatty acids, probiotics etc.	0	0	0
Ergogenic supplements	Dietary supplements containing a concentrated amount of one specific ergogenic substance, e.g., caffeine, creatine, bicarbonate, beta-alanine or nitrate.	1	1	0
Natural products	Herbs, herbs, roots etc.	1	0	0
Multi-ingredient pre-workout supplements	Pre-workout supplements with a blend of (often many) ergogenic and non-ergogenic substances in various concentrations.	3	0	20
Other supplements	Supplements for weight loss, increased libido, hormone modulating supplements, anabolic/muscle building supplements	1	7	7
Unknown^*^		6	1	0
Total		13	9	27

* *The athlete used a combination of specific and not specific products*.

Of the nine cases with a possible association between supplement use and the AAF's, but where a likely causal relationship could not be established with a satisfactory level of probability, seven cases involved Other supplements (muscle building supplements, *n* = 5; fat burning supplement, *n* = 1; products to enhance immune function, *n* = 1), one case involved an ergogenic creatine supplement and one case involved many supplements from various supplement categories. There were no examples of Sport foods, Medical supplements or Natural products containing prohibited substances.

In 15 (56%) of the 27 cases, the product was declared with a prohibited substance corresponding to what was detected in the athlete's urine sample. Products with declared prohibited substances were most common in MIPS supplements, where 14 of 20 cases were declared with the substance methylhexanamine, 1,3-dimethylamylamine (DMAA) or geranium.

Most products were bought in Sweden (*n* = 13 of 27) followed by USA (*n* = 3) and Canada (*n* = 3). The products in the remaining cases were bought from webstores or acquired in physical stores in five different countries (*n* = 5), whereas two athletes got the product from a friend/teammate, and one athlete from his coach.

### Characteristics of Athletes With ADRV's Linked to Supplement Use

The athletes in the supplement ADRV's were predominantly men (93%, *n* = 25 of 27), aged 17–63 years (*Mean* = 26, *SD* = 10). Seventeen (63%) of the 27 cases were of Recreational athletes, while the remaining 10 cases were of NL athletes. There were no analytical ADRV's related to dietary supplements among RTP athletes.

Seventeen of the cases were related to athletes in team sports and 10 cases to individual sports. Among the individual sports, four were strength and power sports, two were VO2max endurance sports, two from other sports, while fighting sports and gymnastic sports each had one case.

## Discussion

This study used quantitative and qualitative data from the website and internal archives of a national anti-doping organization to assess the magnitude of analytical anti-doping rule violations in sports likely related to the use of dietary supplements. By examining 18-years of test statistics and data on anti-doping rule violations (ADRV's), this study provide evidence that the use of dietary supplements containing prohibited substances has been, and still is a major cause of analytical anti-doping rule violations among Norwegian athletes, constituting between 14 and 19% of all analytical ADRV's in the period.

When discussing unintentional doping, one often assume that the athlete accidentally consumed a prohibited substance through food or dietary supplement without any intention to increase their performance (Chan et al., 2019). Unintentional doping following supplement use could occur in multiple ways, for example if the prohibited substance is not declared on the product label, if the prohibited substance is declared, but with another name than what appears in the WADA Prohibited list or when the prohibited substance is declared but the athlete is unaware that the substance is prohibited, and thus assuming the product is safe (Maughan et al., 2018a). There were examples of all these paths to ADRV's following supplement use in the present study.

Supplements with undeclared prohibited substances are either deliberately spiked with prohibited substances by the manufacturer to improve their effectiveness (Mathews, 2018), or cross-contaminated with prohibited substances resulting from poor quality control in the manufacturing, processing or packaging process (Geyer et al., 2008). In the latter case, the amount of the prohibited substance is usually small and thus provide insignificant performance enhancing effects, but could still exceed the cut off for reporting positive doping cases, thus resulting in an analytical ADRV (Catlin et al., 2000; Watson et al., 2009; Duiven et al., 2021).

Dietary supplements declared with a prohibited substance could also pose a threat to athletes if they do not check the specific ingredients prior to consumption against the WADA Prohibited list (Chan et al., 2015; Maughan et al., 2018b; Helle et al., 2019). In this study, 56% of the ADRV's associated with dietary supplements were related to products declared with prohibited substances. However, in several cases, the prohibited substance was listed by another name than what is used in the Prohibited list. For example, it was not uncommon that multi-ingredient pre-workout supplements were labeled with geranium, a plant species, which manufacturers sometimes use as a cover name for the amphetamine-like stimulant methylhexanamine, even though studies have failed to detect methylhexanamine in geranium (Austin et al., 2014). These findings suggest that only checking ingredients on the product label against the WADA Prohibited list may not be adequate to avoid unintentional doping.

Dietary supplements encompass a large heterogenic group of products with a wide range of ingredients and declared effects (Garthe and Maughan, 2018). Even though prohibited substances, such as anabolic steroids and stimulants have been detected in all types of dietary supplements (Geyer et al., 2004, 2008), some product categories can be considered as higher risk than others. This study clearly demonstrates that the risk of a supplement containing a prohibited substance, either declared or non-declared, were not uniformly distributed across all supplement categories. Rather, prohibited substances were mostly confined to multi-ingredient pre-workout supplements, fat burning products and muscle building supplements. There were no associations between ADRV's and the use of sport foods or medical supplements, which are recognized to be the supplement categories most widely used by athletes. These findings are in line with current recommendations advising athletes to avoid or be particularly careful with products containing multiple ingredients and products making claims of performance enhancement or exaggerated claims or uses of the words “stimulant,” “energy booster,” “muscle booster,” “extreme” or “weight loss” (Vernec et al., 2013; Mathews, 2018).

About 60% of the ADRV's associated with supplement use were related to products containing methylhexanamine. Methylhexanamine and similar synthetic stimulants have previously been identified in various multi-ingredient pre-workout supplements (Cohen et al., 2014, 2015) and have been linked to numerous doping cases worldwide (Vernec et al., 2013). Despite of multiple warnings and enforcements actions following deaths and severe adverse health effects (Lieberman et al., 2018), synthetic stimulants are still present in dietary supplements (Cohen et al., 2018; Harty et al., 2018). In this study, the first methylhexanamine ADRV case was registered in 2011 and the latest in 2020.

Previous studies suggest that elite athletes use more supplements than non-elite athletes (Knapik et al., 2016), that supplement use is positively associated with training load (Lun et al., 2012), and are more widely used in speed, power and endurance-based sports than in team sports (Heikkinen et al., 2011). Even though this study does not present prevalence data on supplement use across sports and athlete levels, this is somewhat contradictory to the results from the present study, where a majority of supplement ADRV's were from recreational level athletes and athletes competing in team sports. A possible explanation could be that non-elite athletes and national level athletes in team sports albeit using less supplements, for various reasons more often use high-risk products, such as multi-ingredient pre-workout supplements. As recreational level athletes are seldom doping tested on a regular basis, they are likely less aware of the anti-doping rules, and thus may execute inadequate due diligence when using supplements. Furthermore, it is reasonably to believe that elite athletes and to a lesser degree, national level athletes, more often than lower level athletes have access to a team of coaches, physicians, sport scientists and sport nutritionist, which may provide the athlete with information and guidance on risks of supplement use. More research is needed on supplement use, supplement category and athlete level to get a better understanding of the motivation and individual decision process behind supplement usage across sports and between athlete levels.

Not all supplement ADRV cases are necessarily unintentional doping. Some athletes may want to explain the presence of a prohibited substance in the doping sample by supplement use in an attempt to declare innocence and to avoid sanctions (Whitaker and Backhouse, 2017). In other cases, the athlete had deliberately used a supplement to increase physiological performance for example by trying to reduce body weight or increase exercise intensity. As the athlete's true intentionality for using dietary supplements are difficult to establish with a high level of certainty, the present study does not describe the prevalence of unintentional doping among Norwegian athletes but rather give an estimate of the magnitude of ADRV cases arising from supplement use.

## Conclusion

The only way to eliminate the risk of violation of WADC article 2.1 following dietary supplement use, is to avoid supplements altogether. However, as dietary supplements may be beneficial in certain situations (Maughan et al., 2018b), athletes on all levels are likely to continue using these products.

Anti-doping organizations should develop strategies on how to assist athletes to make informed decision on supplement use (Eichner and Tygart, 2016; Maughan et al., 2018b; Chan et al., 2019), for example by providing knowledge and tools for athletes and support personnel on how to assess the need, assess the risk and assess the consequences of using various supplements (Backhouse et al., 2019; Chan et al., 2020). Furthermore, athletes on all levels should consider decisions around supplement use as a reason to consult a physician, sport nutritionist, sport scientist or any other professional with special expertise in sport nutrition (Maughan et al., 2018a). This study suggests that non-elite athletes in team sports may be a particularly important target group for such interventions. Multi-ingredient pre-workout supplements, muscle building supplements and weight loss supplements should be given special attention, as these products have a high risk of containing prohibiting substances, such as synthetic stimulants and anabolic substances. Athletes should be informed about independent quality assurance programs which screen dietary supplements for prohibited substances (Backhouse et al., 2019) as this will significantly reduce, albeit not eliminate the risk of inadvertent doping following supplement use (de Hon and Coumans, 2007).

As the present data is limited to that of one National anti-Doping organization, the results cannot be used to make generalized conclusions. However, the methods presented here may serve as a template for other anti-doping organizations to follow to get a better understanding of the true magnitude of the problem, between countries, sports and athlete levels.

## Data Availability Statement

The raw data supporting the conclusions of this article will be made available by the authors, without undue reservation.

## Author Contributions

The author has approved the work for publication.

## Funding

The research was funded by an annual grant from the Norwegian Ministry of Culture and Equality to Anti-Doping Norway.

## Conflict of Interest

The author declares that the research was conducted in the absence of any commercial or financial relationships that could be construed as a potential conflict of interest.

## Publisher's Note

All claims expressed in this article are solely those of the authors and do not necessarily represent those of their affiliated organizations, or those of the publisher, the editors and the reviewers. Any product that may be evaluated in this article, or claim that may be made by its manufacturer, is not guaranteed or endorsed by the publisher.

## References

[B1] Austin K. G. Travis J. Pace G. Lieberman H. R. (2014). Analysis of 1,3 dimethylamylamine concentrations in Geraniaceae, geranium oil and dietary supplements. Drug Test. Anal. 6, 797–804. 10.1002/dta.1491 23704033

[B2] Ayotte C. Levesque J. F. Cle roux M. Lajeunesse A. Goudreault D. Fakirian A. (2001). Sport nutritional supplements: quality and doping controls. Can. J. Appl. Physiol. 26(Suppl.), S120–S129. 10.1139/h2001-047 11897888

[B3] Backhouse S. Duiven E. Staff H. Bentley M. (2019). Reducing the Risk of Inadvertent Doping From Food Supplement Use: Current Practice and Future Actions. FAIR Forum for Anti-doping in Recreational Sport. Brussels: EuropeActive.

[B4] Baltazar-Martins G. Brito de Souza D. Aguilar-Navarro M. Munoz-Guerra J. Plata M. D. M. Del Coso J. (2019). Prevalence and patterns of dietary supplement use in elite Spanish athletes. J. Int. Soc. Sports Nutr. 16, 30. 10.1186/s12970-019-0296-5 31319850 PMC6639916

[B5] Baylis A. Cameron-Smith D. Burke L. M. (2001). Inadvertent doping through supplement use by athletes: assessment and management of the risk in Australia. Int. J. Sport Nutr. Exerc. Metab. 11, 365–383. 10.1123/ijsnem.11.3.365 11591885

[B6] Catlin D. H. Leder B. Z. Ahrens B. Starcevic B. Hatton C. K. Green G. A.. (2000). Trace contamination of over-the-counter androstenedione and positive urine test results for a nandrolone metabolite. JAMA 284, 2618–2621. 10.1001/jama.284.20.2618 11086369

[B7] Chan D. K. Donovan R. J. Lentillon-Kaestner V. Hardcastle S. J. Dimmock J. A. Keatley D. A.. (2015). Young athletes' awareness and monitoring of anti-doping in daily life: does motivation matter? Scand. J. Med. Sci. Sports. 25, e655–e663. 10.1111/sms.12362 25441263

[B8] Chan D. K. Ntoumanis N. Gucciardi D. F. Donovan R. J. Dimmock J. A. Hardcastle S. J.. (2016). What if it really was an accident? The psychology of unintentional doping. Br. J. Sports Med. 50, 898–899. 10.1136/bjsports-2015-094678 26392593

[B9] Chan D. K. C. Tang T. C. W. Gucciardi D. F. Ntoumanis N. Dimmock J. A. Donovan R. J.. (2020). Psychological and behavioural factors of unintentional doping: a preliminary systematic review. Int. J. Sport Exerc. Psychol. 18, 273–295. 10.1080/1612197X.2018.1450095

[B10] Chan D. K. C. Tang T. C. W. Yung P. S. Gucciardi D. F. Hagger M. S. (2019). Is unintentional doping real, or just an excuse? Br. J. Sports Med. 53, 978–979. 10.1136/bjsports-2017-097614 28733361

[B11] Cohen P. A. Travis J. C. Keizers P. H. J. Deuster P. Venhuis B. J. (2018). Four experimental stimulants found in sports and weight loss supplements: 2-amino-6-methylheptane (octodrine), 1,4-dimethylamylamine (1,4-DMAA), 1,3-dimethylamylamine (1,3-DMAA) and 1,3-dimethylbutylamine (1,3-DMBA). Clin. Toxicol. 56, 421–426. 10.1080/15563650.2017.1398328 29115866

[B12] Cohen P. A. Travis J. C. Venhuis B. J. (2014). A methamphetamine analog (N,alpha-diethyl-phenylethylamine) identified in a mainstream dietary supplement. Drug Test. Anal. 6, 805–807. 10.1002/dta.1578 24124092

[B13] Cohen P. A. Travis J. C. Venhuis B. J. (2015). A synthetic stimulant never tested in humans, 1,3-dimethylbutylamine (DMBA), is identified in multiple dietary supplements. Drug Test. Anal. 7, 83–87. 10.1002/dta.1735 25293509

[B14] de Hon O. Coumans B. (2007). The continuing story of nutritional supplements and doping infractions. Br. J. Sports Med. 41, 800–805; discussion 5. 10.1136/bjsm.2007.037226 17957017 PMC2465258

[B15] Duiven E. van Loon L. J. C. Spruijt L. Koert W. de Hon O. M. (2021). Undeclared doping substances are highly prevalent in commercial sports nutrition supplements. J. Sports Sci. Med. 20, 328–338. 10.52082/jssm.2021.328 34211326 PMC8219275

[B16] Eichner A. Tygart T. (2016). Adulterated dietary supplements threaten the health and sporting career of up-and-coming young athletes. Drug Test. Anal. 8:304–306. 10.1002/dta.1899 27072842

[B17] Garthe I. Maughan R. J. (2018). Athletes and supplements: prevalence and perspectives. Int. J. Sport Nutr. Exerc. Metab. 28, 126–138. 10.1123/ijsnem.2017-0429 29580114

[B18] Geyer H. Mareck-Engelke U. Reinhart U. Thevis M. Schänzer W. (2000). Positive doping cases with norandrosterone after application of contaminated nutritional supplements. Dtsch. Z. Sportmed. 51, 378–382.

[B19] Geyer H. Parr M. K. Koehler K. Mareck U. Schänzer W. Thevis M. (2008). Nutritional supplements cross-contaminated and faked with doping substances. J. Mass Spectr. 43, 892–902. 10.1002/jms.1452 18563865

[B20] Geyer H. Parr M. K. Mareck U. Reinhart U. Schrader Y. Schanzer W. (2004). Analysis of non-hormonal nutritional supplements for anabolic-androgenic steroids - results of an international study. Int. J. Sports Med. 25, 124–129. 10.1055/s-2004-819955 14986195

[B21] Harty P. S. Zabriskie H. A. Erickson J. L. Molling P. E. Kerksick C. M. Jagim A. R. (2018). Multi-ingredient pre-workout supplements, safety implications, and performance outcomes: a brief review. J. Int. Soc. Sports Nutr. 15, 41. 10.1186/s12970-018-0247-6 30089501 PMC6083567

[B22] Heikkinen A. Alaranta A. Helenius I. Vasankari T. (2011). Use of dietary supplements in olympic athletes is decreasing: a follow-up study between 2002 and 2009. J. Int. Soc. Sports Nutr. 8, 1. 10.1186/1550-2783-8-1 21294857 PMC3042913

[B23] Helle C. Sommer A. K. Syversen P. V. Lauritzen F. (2019). Doping substances in dietary supplements. Tidsskr. Nor. Laegeforen. 139, 334–338. 10.4045/tidsskr.18.0502 30808106

[B24] Jagim A. R. Harty P. S. Camic C. L. (2019). Common ingredient profiles of multi-ingredient pre-workout supplements. Nutrients 11, 254. 10.3390/nu11020254 30678328 PMC6413194

[B25] Knapik J. J. Steelman R. A. Hoedebecke S. S. Austin K. G. Farina E. K. Lieberman H. R. (2016). Prevalence of dietary supplement use by athletes: systematic review and meta-analysis. Sports Med. 46, 103–123. 10.1007/s40279-015-0387-7 26442916 PMC4697915

[B26] Lieberman H. R. Austin K. G. Farina E. K. (2018). Surveillance of the armed forces as a sentinel system for detecting adverse effects of dietary supplements in the general population. Public Health Nutr. 21, 882–887. 10.1017/S1368980017003111 29151367 PMC5848759

[B27] Lun V. Erdman K. A. Fung T. S. Reimer R. A. (2012). Dietary supplementation practices in Canadian high-performance athletes. Int. J. Sport Nutr. Exerc. Metab. 22, 31–37. 10.1123/ijsnem.22.1.31 22248498

[B28] Martínez-Sanz J. M. Sospedra I. Mañas Ortiz C. Baladía E. Gil-Izquierdo A. Ortiz-Moncada R. (2017). Intended or unintended doping? A review of the presence of doping substances in dietary supplements used in sports. Nutrients 9, 1–22. 10.3390/nu9101093 28976928 PMC5691710

[B29] Mathews N. M.. (2018). Prohibited contaminants in dietary supplements. Sports Health 10, 19–30. 10.1177/1941738117727736 28850291 PMC5753965

[B30] Maughan R. J. Burke L. M. Dvorak J. Larson-Meyer D. E. Peeling P. Phillips S. M.. (2018a). IOC consensus statement: dietary supplements and the high-performance athlete. Int. J. Sport Nutr. Exerc. Metab. 28, 104–125. 10.1123/ijsnem.2018-0020 29589768

[B31] Maughan R. J. Shirreffs S. M. Vernec A. (2018b). Making decisions about supplement use. Int. J. Sport Nutr. Exerc. Metab. 28, 212–219. 10.1123/ijsnem.2018-0009 29565185

[B32] Morente-Sanchez J. Zabala M. (2013). Doping in sport: a review of elite athletes' attitudes, beliefs, and knowledge. Sports Med. 43, 395–411. 10.1007/s40279-013-0037-x 23532595

[B33] Outram S. Stewart B. (2015). Doping through supplement use: a review of the available empirical data. Int. J. Sport Nutr. Exerc. Metab. 25, 54–59. 10.1123/ijsnem.2013-0174 25722470

[B34] Petroczi A. Naughton D. P. Mazanov J. Holloway A. Bingham J. (2007). Performance enhancement with supplements: incongruence between rationale and practice. J. Int. Soc. Sports Nutr. 4, 19. 10.1186/1550-2783-4-19 17997853 PMC2214727

[B35] Vernec A. Stear S. J. Burke L. M. Castell L. M. (2013). A–Z of nutritional supplements: dietary supplements, sports nutrition foodsand ergogenic aids for health and performance: Part 48. Br. J. Sports Med. 47, 998–1000. 10.1136/bjsports-2013-092941

[B36] Waddington I. Malcolm D. Roderick M. Naik R. (2005). Drug use in English professional football. Br. J. Sports Med. 39, e18; discussion e. 10.1136/bjsm.2004.012468 15793076 PMC1725181

[B37] Walpurgis K. Thomas A. Geyer H. Mareck U. Thevis M. (2020). Dietary supplement and food contaminations and their implications for doping controls. Foods 9, 1–21. 10.3390/foods9081012 32727139 PMC7466328

[B38] Watson P. Judkins C. Houghton E. Russell C. Maughan R. J. (2009). Urinary nandrolone metabolite detection after ingestion of a nandrolone precursor. Med. Sci. Sports Exerc. 41, 766–772. 10.1249/MSS.0b013e31818edaeb 19276858

[B39] Whitaker L. Backhouse S. (2017). Doping in sport: an analysis of sanctioned UK rugby union players between 2009 and 2015. J. Sports Sci. 35, 1607–1613. 10.1080/02640414.2016.1226509 27578446

[B40] Yonamine M. Garcia P. R. de Moraes Moreau R. L. (2004). Non-intentional doping in sports. Sports Med. 34, 697–704. 10.2165/00007256-200434110-00001 15456345

